# Free-Forging of Pure Titanium with High Reduction of Thickness by Plasma-Carburized SKD11 Dies

**DOI:** 10.3390/ma14102536

**Published:** 2021-05-13

**Authors:** Tatsuhiko Aizawa, Tomoaki Yoshino, Yohei Suzuki, Tomomi Shiratori

**Affiliations:** 1 Surface Engineering Design Laboratory, Shibaura Institute of Technology, Tokyo 144-0045, Japan; 2 Komatsu-Seiki Kosakusho, Co., Ltd., Suwa 392-0012, Japan; yoshino@komatsuseiki.co.jp (T.Y); y-suzuki@komatsuseiki.co.jp (Y.S.); 3 Department of Mechanical Engineering, Faculty of Engineering, University of Toyama, Toyama 930-8555, Japan; shira@eng.u-toyama.ac.jp

**Keywords:** plasma carburizing, SKD11 punch, low temperature, pure titanium wire, cold forging, large deformation, titanium ribbon, in situ solid lubrication, grain size refinement, low residual strains, microtexturing

## Abstract

A tool steel type SKD11 punch was plasma carburized at 673 K for 14.4 ks at 70 Pa to make carbon supersaturation. This carburized SKD11 punch was employed for upsetting the pure titanium wire with the diameter of 1.00 mm up to the reduction of thickness by 70% in a single shot. Its contact interface to titanium work was analyzed to describe the anti-galling behavior in this forging. Little trace of titanium proved that the galling process was suppressed by the in situ solid lubrication. The isolated free carbon agglomerates are wrought as a solid lubricant to sustain the galling-free forging process. This anti-galling upsetting reduced the residual strains in the forged wires. A long titanium wire with a length of 45 mm was incrementally upset to yield the titanium ribbon with a thickness of 0.3 mm, the width of 2.3 mm, and the length of 50 mm. The grain size of original pure titanium was much reduced to 2 μm on average. A micro-pillared microtexture was imprinted onto this forged titanium ribbon.

## 1. Introduction

As a die material for cold forging, WC (Co) and monolithic ceramics have been utilized in industries but often suffered from the severe galling or work mass transfer onto the die contact interface in cold, dry forging of titanium and titanium alloys [1,2]. In addition to the adhesion of metallic titanium fragments, the titanium oxide debris particles splashed in air and deposited onto the die surface. No improvement in drawability was experienced even when using ZrO_2_, SiC and Si_3_N_4_ die materials in deep drawing of titanium sheets. As surveyed in [3], these galling processes were difficult to be prevented from upsetting and shaping the pure titanium works with high reduction of thickness without an innovative change of die materials. Thick *β*-SiC coated SiC die material was the first candidate to put the anti-galling forging in dry and cold into practice [4,5,6]. Those chemical vapor deposition (CVD)-coated *β*-SiC layers had stable 3C-structure with supersaturated carbon solute contents. A pure titanium work was continuously upset up to the reduction of thickness by 35% without metallic titanium transfer to *β*-SiC coating [4]. The carbon agglomerates isolated from the carbon supersaturated *β*-SiC coating and wrought as a solid lubricant together with low friction by the in situ formed deformable intermediate titanium oxide tribofilm [5]. Due to this in situ solid lubrication, the titanium wire with a diameter of 1.0 mm was reduced to a rectangular plate with a thickness of 0.3 mm by a single-shot upsetting [6]. As surveyed in [7], this in situ solid lubrication by the free carbon dots and tribofilms plays a role in the die material selection to make galling-free, dry, cold forging in practice.

The second candidate of anti-galling die materials for cold dry forging was low-temperature plasma-carburized stainless steels [7,8]. AISI420J2 substrate was employed to make carbon supersaturation by plasma carburizing at 673 K. The carburizing time was 14.4 ks or 4 h. In situ formation of unbound carbon film worked as a solid lubricant to prevent the AISI420J2 punch from severe adhesion of titanium fragments and debris particles. This pretreatment of die materials was also effective to austenitic stainless steels, as reported in [9,10,11]. However, very few studies were found with respect to the low-temperature plasma carburizing of tool steel dies. In the die material selection, how to make full use of SKD11 and SKH51 tools is still an issue for improvement of the cost-competitive performance in practice.

In addition, the workability in forging is expected to improve by using the plasma-carburized SKD11 dies. At first, the residual strains are reduced by low friction and low adhesion onto the contact interface of the punch to the work material. As suggested in [12], the warm and cold forging processes are preferable for much reducing the internal strains and homogeneously lowering the hardness profile. In second, the grain size is also reduced by galling-free upsetting. In the literature, the metal forming with a high reduction of thickness often accompanies microstructure evolution and grain size refinement. In [13], a high-pressure torsion was applied to a pure aluminum work to significantly refine its grain size from the order of 10 μm to sub-microns. An intense rolling was also applied to low carbon steel for the reduction of grain size and crystallographic evolution with the reduction of thickness [14]. This approach was also available to yield the homogeneously fine-grained austenitic stainless steel wires from the initial round bar for medical usage [15]. In particular, a demand for miniature biomedical tools in endoscope operation requires fine-grained titanium and titanium alloy material with high strength and hardness and without loss of ductility and toughness [16]. As noticed in [17], the previous studies concentrated on the development of a new alloying design to promote mechanical properties. Aiming at the biomedical wires with higher performance, intense forging and rolling were also employed to refine the initial grain size, together with thermal annealing and heat treatment [18].

In the present paper, a JIS SKD11 (or AISI/SAE D3) punch is first plasma carburized at 673 K for 14.4 ks (4 h) to describe the carbon supersaturation process by X-ray diffraction (XRD), scanning electron microscopy (SEM), and electron dispersive X-ray spectroscopy (EDX). A computer numerical control (CNC) stamping system is employed to make dry and cold forging of a pure titanium wire with a high reduction of thickness. A short wire with a diameter of 1.00 mm and a length of 10 mm is upset in a single shot to a flat plate with a thickness of 0.3 mm. A longer wire with a diameter of 1.00 mm and a length of 45 mm is incrementally forged to fabricate a titanium ribbon with a thickness of 0.3 mm and a length of 50 mm. A contact interface of plasma-carburized SKD11 punch in these forging processes is also analyzed by SEM–EDX to demonstrate that unbound carbon solute isolates from the carbon supersaturated SKD11 punch and agglomerates to a tribofilm for in situ solid lubrication. The grain size refinement by uniform upsetting with high reduction of thickness by 70% is also investigated to search for the possibility to make secondary forming of the titanium ribbon to miniature products with high quality. A microtexture is imprinted onto this grain-refined titanium ribbon to have cubic micropillars with a square head of 125 μm × 125 μm.

## 2. Experimental Procedure

Low-temperature plasma carburizing system was introduced with comments on the experimental setup. A CNC stamping system (Zenformer MPS404; Hoden-Seimitsu, Co., Ltd.; Kanagawa, Japan), was used for cold dry forging with the use of plasma-carburized SKD11 punch. Two forging setups were employed to demonstrate the anti-galling reduction of titanium wires to flat plates, e.g., a single-shot forging and an incremental forging with a high reduction of thickness.

**Figure 1:**
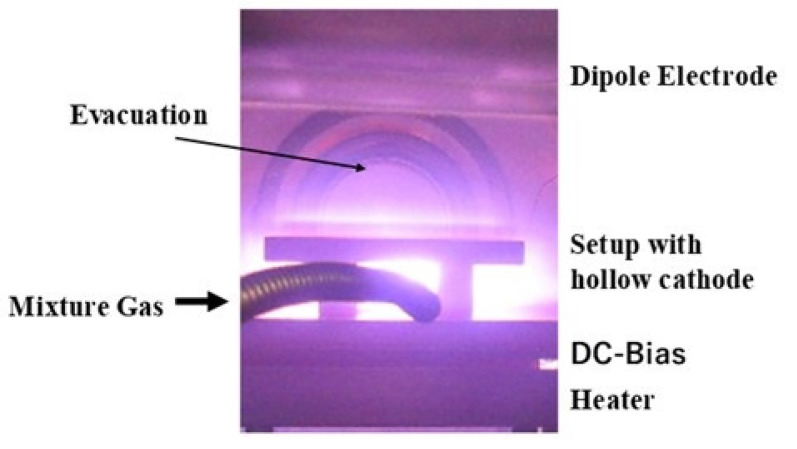
RF–DC plasma carburizing system with the use of hollow cathode device to intensify the ion and radical densities.

Although the direct current (DC) plasma facilities were utilized in the previous studies [9,10,11], a radio frequency (RF)–DC plasma carburizing system (CVD-I, YS-Electric Industry, Co., Ltd., Koufu, Japan) was employed in this study with the use of the hollow cathode device, as illustrated in Figure 1.

In the following experiments, a mixture gas of argon and hydrogen with the flow rate ratio of 100 mL/min to 80 mL/min was introduced to a chamber for presputtering after the chamber was evacuated, filled with argon, and heated to the holding temperature of 673 K. This presputtering for 1.8 ks (0.5 h) at 673 K in 70 Pa was followed by the plasma carburizing at 673 K, in 70Pa for 14.4 ks (4 h) after introducing CH_4_ gas by 20 mL/min. In this carburizing process, RF–voltage and DC–bias, were constant by 200 V and −600 V, respectively. After carburizing, the chamber was evacuated and cooled down in a nitrogen atmosphere. The resulting carburized layer thickness was 45 μm.

### 2.1. CNC Forging System

**Figure 2:**
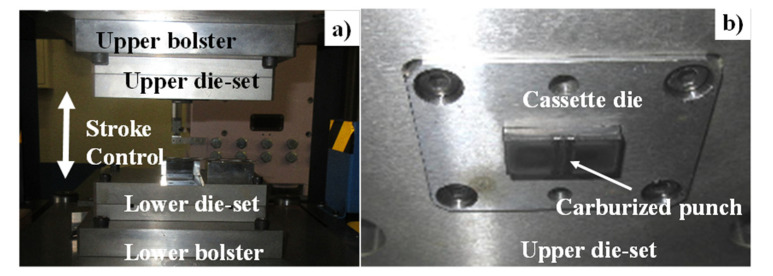
CNC stamping system: (**a**) CNC stamper for cold forging and (**b**) upper cassette die with the plasma-carburized SKD11 punch.

The CNC stamper with the maximum loading to 50 kN, was utilized to make dry cold forging experiments with the use of plasma-carburized SKD11 punch, as depicted in Figure 2a. The plasma-carburized punch was placed into a cassette die, which was further cemented into the upper die set, as depicted in Figure 2b.

### 2.2. Single-Shot and Continuous Forging Steps with Secondary Forging

Two types of cold, dry forging experiments were performed with the use of plasma-carburized SKD11 punch. In the single-shot forging, the short titanium wire with the diameter of 1.00 mm and the length of 10 mm was upset by varying the reduction of the thickness (r) to describe the forging behavior of titanium without galling. Microstructure evolution by high reduction forging is also described by this experiment. Microhardness testing was utilized to measure the hardness distribution on the cross section of each forged titanium wire. In addition, SEM and electron back-scattering diffraction (EBSD) systems (JOEL, Tokyo, Japan) were also employed to describe the in situ grain size refinement during the upsetting. A long wire with a diameter of 1.00 mm and a length of 45 mm was incrementally forged to shape a titanium ribbon with a thickness of 0.3 mm. The forging velocity was constant by 0.1 mm/s. The power–stroke relationship was in situ measured as had been reported in [8].

As the secondary forging step, a mesh-patterned SKD11 punch was utilized to imprint the micropillars onto this titanium ribbon. The unit microcavity of this mesh-patterned punch was shaped to a regular box cell with 125 μm × 125 μm × 200 μm. This punch was fabricated by the plasma printing method [19].

### 2.3. Punch and Work Materials

A tool steel type SKD11 (or AISI/SAE D3) was employed as a punch material for plasma carburizing and forging. Its chemical composition was as follows: carbon by 1.44 mass%, silicon by 0.3 mass%, manganese by 0.35 mass%, phosphorous by 0.27 mass%, sulfur by 0.06 mass%, chromium by 11.1 mass%, molybdenum by 0.8 mass%, vanadium by 0.2 mass%, and iron in balance. A pure titanium type TP328H in industrial grade II was employed as a work material in the forging experiments. Its chemical composition consists of hydrogen by 0.0012 mass%, oxygen by 0.097 mass%, nitrogen by 0.007 mass%, iron by 0.042 mass%, carbon by 0.007 mass%, and titanium for balance.

## 3. Experimental Results

### 3.1. Carbon Supersaturation into SKD11 Dies by Plasma Carburizing

**Figure 3:**
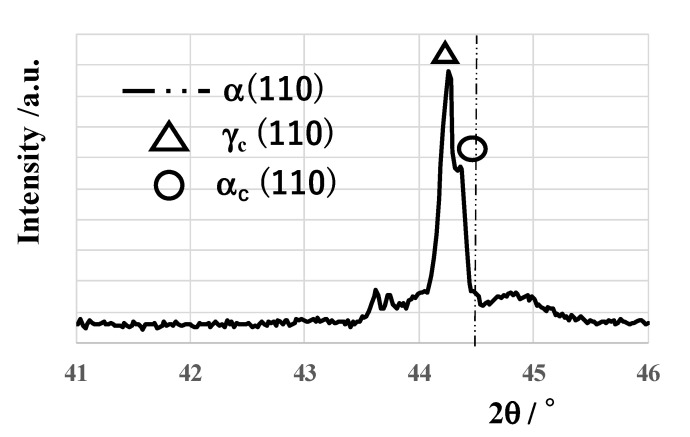
XRD diagram on the plasma-carburized SKD11 at 673 K for 14.4 ks with comparison to a bare SKD11 before carburizing.

XRD (D8, Burker, Toyama, Japan) was utilized to analyze the plasma-carburized SKD11 punch surface. As depicted in Figure 3, the original α-iron peak (110) shifted to the lower 2θ side. The original bcc (body-center cubic) structure expands itself by the carbon supersaturation. In addition to this peak shift, the expanded austenitic phase is also detected in Figure 3. As reported in [8,20], the austenitic phase transformation takes place with the carbon supersaturation into SKD11.

Due to this carbon supersaturation, SKD11 punch is also hardened to have an average surface hardness of 1200 HV.

### 3.2. Single-Shot Forging of Short Titanium Wire

**Figure 4:**
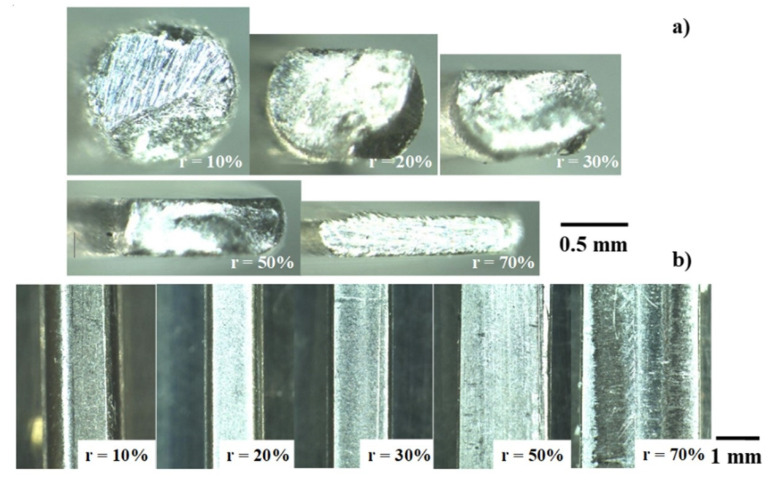
Variation of the forged pure titanium wire with the diameter of 1.00 mm and the length of 10 mm with increasing the reduction of thickness (r): (**a**) cross sections of forged wire and (**b**) its plain view.

Figure 4a depicts the variation of wire cross section with r. When r < 30%, a wire is axially compressed to reduce its diameter. For r > 30%, it is laterally flattened and shaped into a flat plate. As shown in Figure 4b, the total width of wire (Wo) and the width of contact interface (Wc) broaden with r. This Wc significantly increases with r so that (Wo-Wc) reduces and goes to nearly zero for r > 30%. Since the bulging deformation (=(Wo − Wc)/2) monotonously reduces with r, the flow velocity in the lateral direction becomes uniform with less barreling deformation.

In general, a large bulging deformation is noticed as a barrel when upsetting the wires and bars with a higher friction coefficient, while this bulging deformation is reduced by upsetting them with lower friction so that their lateral plastic flow velocity becomes uniform and homogeneous [21]. In Figure 4, (Wo − Wc)~0 or less bulging deformation takes place for r > 30%. This implies that the friction coefficient is reduced down to 0.05 to 0.1 on the contact interface of plasma-carburized SKD11 punch to titanium work. As reported in [1], a bare SKD11 punch and die suffered from severe galling when upsetting the titanium and titanium alloy wires, even at r = 30%. This difference in the forging behavior between bare- and plasma-carburized SKD11 punches comes from the tribological mechanism on the contact interface of punch to the forged titanium work.

### 3.3. Hardness Profile on the Cross Section of Forged Titanium Wire

The upsetting and forging processes with a high reduction in thickness also suffered from the high residual strains by the work hardening of metallic works and shear band formation. In particular, under a high frictional state on the contact interface by galling, this hardening hinders further upsetting and forging without annealing the work materials.

**Figure 5:**
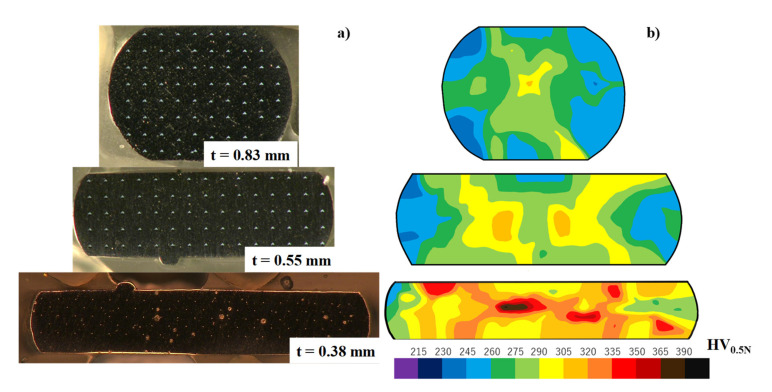
Variation of the cross-sectional shape and the hardness profile on the cross sections with increasing the reduction of thickness: (**a**) variation of the cross sections with r and (**b**) variation of the hardness profile on the cross section with r.

The micro-Vickers hardness testing was employed to describe the evolution of hardness mapping on the cross section of upset wires at the reduced thickness (t). As shown in Figure 4a and Figure 5a, the original wire with the diameter of 1.00 mm was incrementally upset down to the plate with a thickness of 0.38 mm. Micro-Vickers hardness was measured on each cross section of upset wire regularly with the pitch of 100 μm. Figure 5b shows the hardness mapping on each cross section at t = 0.83 mm, 0.55 mm, and 0.38 mm, respectively. When r = 45%, most of the cross section has 320 HV_0.5N_ in maximum and 280 HV_0.5N_ on average. Different from the normal inner straining with the shear band during upsetting with high reduction of thickness, the inner strain distribution becomes uniform with less strain concentration or shear bands. This reveals that no thermal annealing is necessary even when r exceeds 50% in thickness. In the hardness mapping at r = 62%, most of the cross section has uniform hardness around 300 HV_0.5N_, excluding the center part of the wrought wire. This proves that the anti-galling upsetting process accompanies low residual straining, even in high reduction of thickness, and becomes feasible to the first step in the precise forging procedure to the final product.

### 3.4. Microstructure Analysis on the Contact Interface

**Figure 6:**
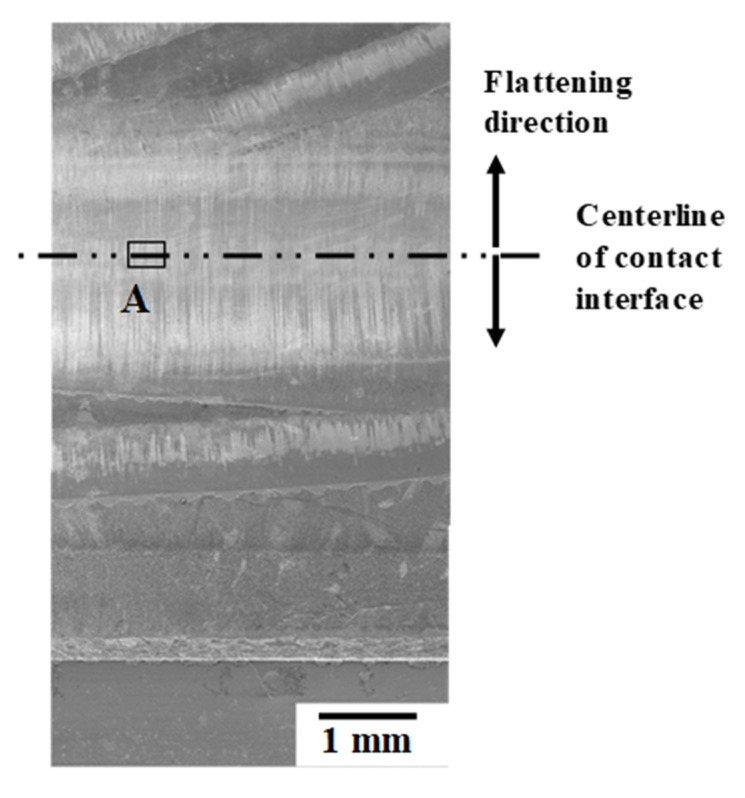
Optical microscopy observation on the contact interface of the carbon supersaturated SKD11 punch to the wrought titanium wires.

The low friction and low internal straining are attributed to the in situ lubricating condition on the contact interface of carbon supersaturated SKD11 to the titanium wires. SEM–EDX analysis (JOEL, Tokyo, Japan) was employed to describe the formation of tribofilm on the contact interface. Figure 6 shows an optical microscopic image on the contact interface of plasma-carburized SKD11 punch to forged titanium wires after continuously forging 15 shots with r = 70%. This tribofilm works until the carburized punch has fatal damage during continuous forging processes. On the contact interface, the traces are formed in stripes from its centerline to its end. A-region at the centerlines was selected in Figure 6 for SEM–EDX analysis.

**Figure 7:**
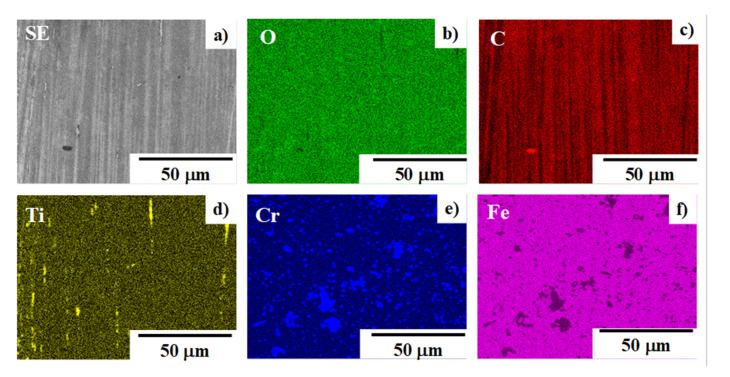
SEM–EDX analysis on the centerline of the contact interface: (**a**) SEM image and (**b**–**f**) element mapping by oxygen carbon, titanium, chromium, and iron, respectively.

In correspondence to the optical microscopic image in Figure 6, the center part of the contact interface all consists of the dense stripes radially aligned with each other, as depicted in Figure 7a.

Element mapping of oxygen, carbon, titanium, chromium, and iron is, respectively, shown in Figure 7b–f. Both the chromium and iron mapping have no correlation to stripe formation in Figure 7a. Titanium and oxygen lines are noticed in their mapping but have less correlation. Only carbon mapping is just corresponding to this stripe formation. This reveals that carbon stripes are radially formed on the contact interface in correspondence to the plastic flow of titanium wire in the lateral direction, as seen in Figure 4. Remember that the titanium wire was continuously upset 15 times down to r = 70% by a single shot. Each carbon stripe is randomly formed on the contact interface in each upsetting process, resulting in the dense carbon stripe structure. Nearly zero trace of metallic titanium is detected on the contact interface except for the titanium oxides. This implies that no galling takes place in the presence of carbon stripes on the interface. In situ solid lubrication works in every upsetting process to attain low friction and less adhesive wear by preventing the punch surface from galling pure titanium.

### 3.5. Continuous Forging of Long Titanium Wire with the Constant Reduction of Thickness by 70%

**Figure 8:**
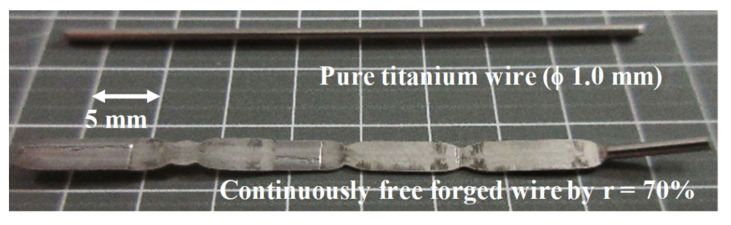
Comparison between a bare titanium wire with a diameter of 1.0 mm and a length of 45 mm and a forged titanium ribbon with a thickness of 0.3 mm and a length of 50 mm.

A titanium wire with a diameter of 1.00 mm and a length of 45 mm was employed to convert the wire to a ribbon with a thickness of 0.3 mm and a length of 50 mm by upsetting with the reduction of thickness by 70%. Figure 8 compares a raw titanium wire and a forged titanium ribbon by successive forging with the use of plasma-carburized SKD11 punch.

A uniform ribbon is yielded by the present upsetting with high reduction even without additional treatments and lubrications. Small necking points are regularly seen in every length of 10 mm, which is equivalent to the width of the punch. The titanium wire in contact with both ends of the punch cannot deform in the radial direction.

**Figure 9:**
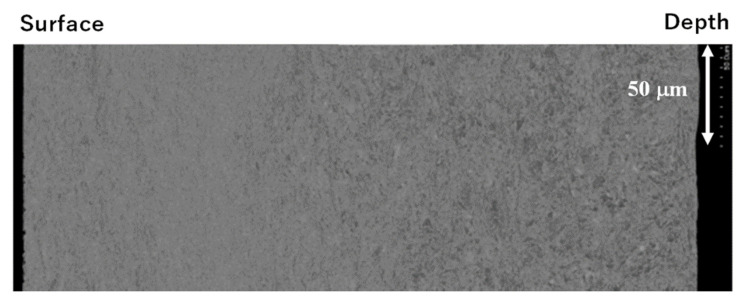
SEM analysis on the cross section of continuously free-forged pure titanium ribbon, as shown in Figure 8.

The present anti-galling upsetting process with a higher reduction of thickness than 70% also provides a useful method to refine the grain size for precise shaping. Figure 9 depicts the SEM image from the surface to the depth of the wrought titanium wire cross section at r = 70%.

**Figure 10:**
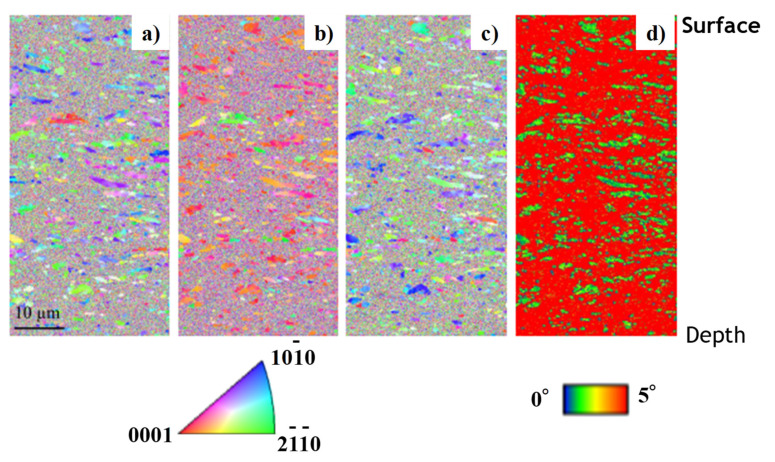
EBSD analysis on the cross section of continuously free-forged pure titanium ribbon at region A. (**a**) IPF profile in ND, (**b**) IPF profile in RD, (**c**) IPF profile in TD, and (**d**) KAM distribution.

The original grain size of 25 μm on average was refined down to 2 μm, especially at the vicinity of the surface. The inverse pole figures (IPF) in the normal direction (ND), the rolling direction (RD), and the tangential direction (TD) are also shown in Figure 10, together with the Kernel average misorientation (KAM). The intrinsic tissues in the titanium wire by rolling and extrusion are still left in the matrix. Most of the matrices are modified to have fine grain sizes with large crystalline misorientation. Figure 8 and Figure 9 prove that grain size refinement takes place together with upsetting the titanium wire in high reduction of thickness.

### 3.6. Microtexturing into the Continuously Free-Forged Pure Titanium Ribbon

As the secondary process to the above continuous free-forging of the pure titanium wire, the wrought titanium ribbon is further microtextured to have square pillars on the surface. These micropillars are formed as an anchor of titanium spikes and as an interface to fix the titanium plate to cells in operation. The plasma nitrided AISI316 punch with meshing patterned head [19] was utilized for this microtexturing. This punch had multi-meshing heads with their line width of 50 μm and pitch of 175 μm. A unit cell of this punch head between two adjacent mesh lines becomes a microcavity with a square inlet of 125 μm × 125 μm and a depth of 200 μm.

**Figure 11:**
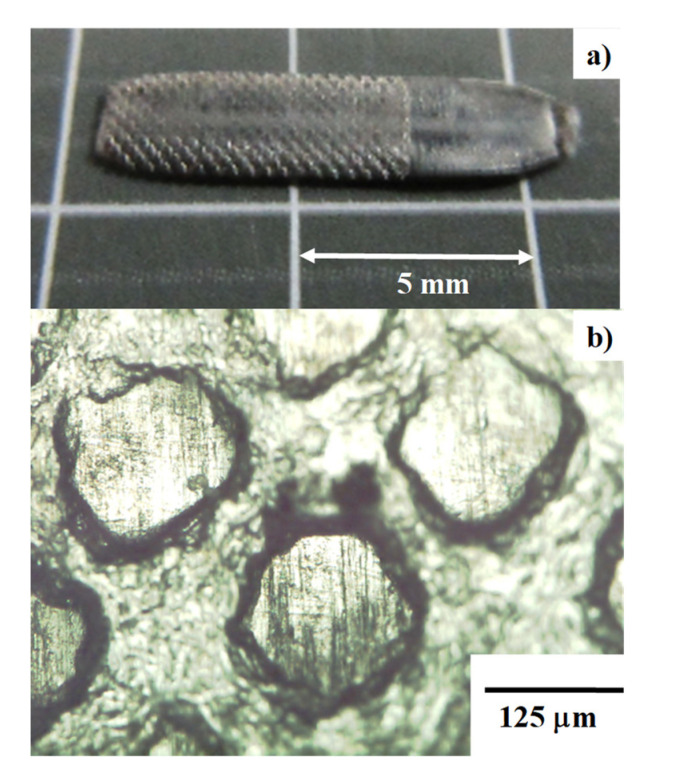
Imprinting of the micropillars onto the continuously free-forged pure titanium ribbon by using the microtextured punch: (**a**) overview of a microtextured titanium plate and (**b**) a micropillared surface with a regular alignment of micropillars with their head of 125 μm × 125 μm and height of 70 μm.

Figure 11a shows the micro-textured titanium plate with the original thickness of 0.3 mm. Its left half has a micro-pillared surface, while its right half is an original wrought titanium surface in Figure 8. The microcavity alignment on the meshing-patterned punch head is imprinted into the micropillar alignment on the microtextured titanium plate surface.

## 4. Discussion

A solid lubricant such as MoS_2_ and graphitic carbon has been widely utilized in the cold and hot forming of metals and metallic alloys in industries [22,23]. Since these solid-lubricating materials have a low dimensional crystalline structure, low friction and wearing conditions are maintained by their self-shearing process, especially in hot conditions. If they were present on the hot spot in the contact interface to trigger the galling, that solid lubrication could be effective to be from galling. However, those solid lubricants could have no role in lubrication if they were absent on the hot spot. As seen in Figure 6 and Figure 7, the carbon solutes isolate from the supersaturated matrix of punch onto the highly stressed zones in the contact interface and form the lubricating films from the centerline to the end of the interface. This self-lubrication is effective to sustain the anti-galling forging process with continuous usage of the carbon supersaturated SKD11 punch.

When using the SiC-coated SiC dies and the *β*-SiC coating dies, this in situ solid lubrication process is common to the present system with the use of carbon supersaturated SKD11. In those SiC dies, the isolated carbon formed the solid lubricating dots and agglomerates, while the carbon stripes are formed radially from the centerline to the end of the contact interface in the present study. With a comparison of the solid lubrication to the carburized AISI420J2, these carbon stripes are formed more homogeneously on the contact interface. This homogeneous, in situ solid lubrication reflects on the low friction and low residual straining, as observed in Figure 4 and Figure 5, respectively.

As reported in [1,24], when using the cemented carbide and tool steel dies for upsetting, the reduction of titanium work thickness was limited by 20 to 30% in each step of upsetting at the risk of galling to die surfaces. Especially, the splash and deposition of TiO_2_ debris particles onto the die surfaces often have a risk to lock the incremental forging and to damage the die surfaces. As shown in Figure 4, a titanium wire is upset in a single shot down to 70%. In addition, this reduction of thickness is governed by the loading capacity of the CNC stamper in the present study. If this capacity were promoted to compensate for the work hardening of forged titanium wires, a much higher reduction of thickness could be attained without any change in the forging process.

Every successive step during upsetting and forging with high reduction accompanies the accumulation of residual internal strains in work materials. In particular, these internal strains are enhanced by the friction and galling on the contact interface to the punch and die. As shown in Figure 5, a relatively homogeneous hardness profile on the cross sections of wrought wire reveals that accumulation of internal strains is suppressed even in high reduction upsetting by using the in situ lubricated SKD11 punch. Since no shear bands are formed on the cross sections of the wire, the successive shaping from the raw wire to the titanium product can be performed with a minimum thermal annealing step.

As seen in Figure 8, the upsetting with high reduction of thickness works as a preliminary step to convert the raw titanium and titanium alloy wire and bar to its ribbon with the thin sheet thickness by (1–r) times of their initial diameter in a similar manner to continuous rolling process [25]. As depicted in Figure 9 and Figure 10, the in situ grain size refinement and homogenization are preferable to the fabrication of medical titanium and titanium alloy wires and tools. The grain size refinement and crystallographic control are performed by these preliminary upsetting to prepare for the secondary steps in precise shaping to the medical products.

Even before COVID19 infection prevention policies, the micro-/nanotexturing methods to metallic and polymer biomedical parts and tools were highlighted to promote antibacterial performance [26]. A precise forging with the use of plasma-printed punch is effective to control the micro-/nanotexture pattern and its unit-cell size on the titanium and titanium alloy tools. In addition, fine nanotextures can be imprinted onto the metallic part and tool surfaces by using the laser-printed SKD11 punch and die, as reported in [27]. Different from the nanostructured titanium oxides in [26], the micro-/nanotextured titanium and titanium alloy parts and tools are available by utilizing the present approach.

## 5. Conclusions

The plasma-carburized SKD11 die technology is developed to make galling-free forging of pure titanium. The free carbon solute isolates from the vicinity of carbon supersaturated SKD11 punch surface, and, in situ forms the carbon tribofilms on the contact interface of SKD11 punch to the titanium work. Due to this in situ solid lubrication, a higher reduction of thickness than 50% is attained in a single shot of upsetting to yield the pure titanium plates and ribbons from their raw wires and bars. No shear bands are generated on the cross section of wires and bars by this upsetting. The residual inner straining is sustained to be low and homogeneous in the cross sections so that no thermal annealing is needed to make the further steps in forging.

Together with high reduction forging, the grain size refinement takes place to transform the normal grain size of 25 μm on average down to fine grains with the size in the order of micron and submicron. These fine-grained pure titanium plates are useful as a starting work material for the second step to form the micro-/nanotextures onto products. In particular, the present approach is adaptive to the fabrication of biomedical titanium and titanium alloy parts and tools with antibacterial surface quality.

## Data Availability

Not applicable.
